# Vitamin D Deficiency and Gestational Diabetes Mellitus in Relation to Body Mass Index

**DOI:** 10.3390/nu14010102

**Published:** 2021-12-27

**Authors:** Nuria Agüero-Domenech, Silvia Jover, Ana Sarrión, Javier Baranda, José A. Quesada-Rico, Avelino Pereira-Expósito, Vicente Gil-Guillén, Ernesto Cortés-Castell, María J. García-Teruel

**Affiliations:** 1 Department of Gynaecology and Obstetrics, Hospital General Universitario Elda, 03600 Elda, Spain; nuria.aguero@umh.es (N.A.-D.); jover_sil@gva.es (S.J.); sarrion_ana@gva.es (A.S.); baranda_jav@gva.es (J.B.); garcia_mjter@gva.es (M.J.G.-T.); 2 Department of Public Health, History of Science and Gynaecology, Miguel Hernández University, 03550 San Juan, Spain; 3 Department of Clinical Medicine, Miguel Hernández University, 03550 San Juan, Spain; vgil@umh.es; 4 Research Unit, Hospital General Universitario Elda, 03600 Elda, Spain; pereira_ave@gva.es; 5 Department of Pharmacology, Pediatrics and Organic Chemistry, Miguel Hernández University, 03550 San Juan, Spain; ernesto.cortes@umh.es

**Keywords:** gestational diabetes mellitus, 25(OH)D concentration, vitamin D deficiency, body mass index, pregnant women

## Abstract

A relationship between vitamin D deficiency (VDD) and gestational diabetes mellitus (GDM) has been described. Considering that GDM prevalence depends on body mass index (BMI), our main objective was to determine if VDD is associated with GDM, independent of BMI. A cross-sectional study with 886 pregnant women was conducted in Elda (Spain) from September 2019 to June 2020. To assess the association, Poisson regression models with robust variance were used to estimate the prevalence ratio (PR). The observed GDM prevalence was 10.5%, while the VDD prevalence was 55.5%. In the crude model, both VDD and obesity were associated with GDM, but in the adjusted model, only VDD was statistically significant (PR = 1.635, *p* = 0.038). A secondary event analysis did not detect differences in VDD, but BMI yielded a higher frequency of births by cesarean section and newborns with a >90 percentile weight in the obesity group. In conclusion, VDD is associated with GDM, independent of BMI. Future longitudinal studies could provide information on causality.

## 1. Introduction

In adults, vitamin D deficiency (VDD) is defined as a 25-hydroxivitamin D (25(OH)D) serum level below 20 ng/mL and vitamin D insufficiency between 20 and 30 ng/mL [1]. There is recent evidence that VDD is a reality both in Spain and globally [2,3,4]. VDD is found in 40.4% of the general population in Europe and 26.0% in the United States of America [5]. Vitamin D levels are similar in the Spanish and European populations [6]. VDD has been described in all ages and genders, in a similar fashion around the globe, including in very sunny regions in Mediterranean countries [7].

Globally, VDD is found in 54% of pregnancies and in 75% of newborns. This affects pregnant women of all latitudes, not only those with lower exposure to sunlight. In Europe, the VDD prevalence in pregnancy is 57%, while it is 73% in newborns [8]. Despite the mentioned high frequency of VDD, there is no consensus about the need for 25(OH)D level evaluation or the requirement of supplementation in pregnancy [9].

VDD has been linked to the occurrence of several adverse events during pregnancy, such as preeclampsia, gestational diabetes mellitus (GDM), preterm birth, and birth by cesarean section. Consequences in newborns include a low weight at birth, a small head circumference, and neurodevelopmental problems [9,10,11]. A recent systematic review compared vitamin D supplementation with a placebo/control and found a reduction in the relative risk of preeclampsia, GMD, and low weight at birth (<2500 g). However, no difference in the risk of preterm birth (<37 weeks) was detected [9].

GDM characterizes a population of women at high risk of developing type 2 diabetes mellitus, representing an early stage in the natural history of the disease [12]. It has been postulated that vitamin D could be key to hepatic metabolism regulation, function, and the development of pancreatic islands, calcium levels in the blood, oxidative stress, and the immune and inflammatory systems that mediate the start of GDM [13]. VDD could be an independent cardiovascular risk factor and a marker of metabolic syndrome [14], as per other existing biomarkers of metabolic syndrome and insulin resistance, such as “homeostasis model of assessment—insulin resistance” (HOMA-IR), levels of fasting insulin, fasting glycemia, C-reactive protein, fibrinogen, and lipid profile, among others [15].

GDM is a highly prevalent disease, with a frequency of 7–12% in the Spanish pregnant women population [16]. This prevalence is associated with body mass index (BMI), with a frequency of 10.2% for a BMI > 25 and 16.7% for a BMI > 30 [17].

Our main objective was to determine if VDD is associated with GDM, independent of BMI. In addition, the secondary endpoint was whether VDD and BMI are associated with a higher occurrence of secondary obstetric or neonatal events.

## 2. Materials and Methods

### 2.1. Study Population

We performed a population-based, single-center, observational, cross-sectional, and analytic study. To achieve the secondary endpoint, a longitudinal follow-up was completed for all participating women, until the completion of pregnancy. All pregnant women in the Health Department were invited to participate. The study was approved by the Institutional Review Board of the Hospital General Universitario Elda (protocol code VITD), and all participants signed an informed consent form prior to inclusion.

Exhaustive consecutive sampling was carried out from September 2019 to July 2020, with the only inclusion criterion being the state of gestation in the second trimester of pregnancy. Multiple pregnancies and a personal history of pregestational diabetes mellitus (type 1 or 2) were considered as exclusion criteria.

### 2.2. Data Collection

Data were collected in a 10-month period via anonymized data sheets, in a retrospective and prospective way, depending on the variable.

#### 2.2.1. Assessment of Vitamin D Levels

Blood samples were taken during the routine visit in the second trimester, in conjunction with a GDM screening test. The 25(OH)D serum concentration was determined by an automated electro-chemiluminescent binding assay (Modular Analytics E170 and Elecsys Vitamin D Total II, Roche Diagnostics, GmBH (Manheim, Germany)), with a measurement range from 3 to 70 ng/dL. This test has previously been validated against liquid chromatography–tandem mass spectrometry (LC/MS/MS), and it has been certified by the CDC Vitamin D Standardization-Certification Program (VDSCP) [18,19,20,21,22].

VDD was defined as a serum concentration <20 ng/mL [1,23]. In the case of VDD, 480 UI of calcidiol per day was supplemented until the end of pregnancy.

#### 2.2.2. Assessment of GDM

GDM diagnosis was completed in two steps, following the Carpenter and Coustan criteria [24] as proposed in the 4th Workshop-Conference on Gestational Diabetes Mellitus [25] and by the American Diabetes Association [26]. A positive screening test (O’Sullivan test) was followed by an oral glucose tolerance test (OGTT) with 100 g of glucose, assessing 0 h, 1 h, 2 h, and 3 h glycemia. If ≥ 2 values were higher than the cut-off value at any time of the OGTT (glycemia cut-off points: 0 h > 95 mg/dL, 1 h > 180 mg/dL, 2 h > 155 mg/dL, and 3 h >140 mg/dL), the diagnosis of GDM was established. These criteria have been previously validated in the Spanish population [27].

#### 2.2.3. Assessment of BMI

BMI was calculated from the weight (in kg) and height (in m) measurements registered in the routine visit of the first trimester of pregnancy. According to BMI, the women were classified as normal (<25), overweight (25–30), and obese (>30), following the World Health Organization’s classification [28].

#### 2.2.4. Assessment of Covariates

In the first-trimester control visit, medical history and physical evaluation were completed, collecting sociodemographic and pregnancy-related data, including maternal age (in years), smoking habit, hypothyroidism, ethnicity, weight (in kg), height (in m), parity, and previous history of cesarean section.

In the second trimester, coinciding with the 20-week ultrasound examination, a survey was filled out (St. Carlos Study questionnaire) to assess physical activity, nutritional habits, and lifestyle, presented as scores. This questionnaire is based on the American Diabetes Association (ADA) evidence-based nutrition recommendations, adapted to the Spanish population following the Diabetes Nutrition and Complications Trial (DNCT), and it has been validated in our population [29,30]. The routine second trimester blood test included: C-reactive protein (CRP) (in mg/dL), ferritin (in mg/dL), total cholesterol (in mg/dL), HDL cholesterol (in mg/dL), LDL cholesterol (in mg/dL), triglycerides (in mg/dL), fibrinogen (in mg/dL), hemoglobin (in g/dL), and hematocrit (in %).

After the delivery, all of the other variables studied were collected: gestational hypothyroidism, preterm birth, preeclampsia, birth by cesarean section, newborn admission to the intensive care unit (ICU), and newborn weight, height, and head circumference percentiles (based on Spanish growth charts).

### 2.3. Statistical Analysis

A descriptive analysis explored the distribution of the study variables, with frequency calculations for categorical variables and mean and standard deviations for continuous variables. A pairwise methodology was employed for missing data management. For the sociodemographic variables, the 95% confidence intervals were calculated with the Wilson method for proportions and the asymptotic method for continuous variables. Factors associated with GDM and VDD were analyzed with contingency tables. The qualitative variables were compared using the chi-square test, and Student’s *t*-test was used for quantitative variables.

To estimate the magnitude of associations with GDM, crude and adjusted Poisson regression models with robust variance were developed, and the prevalence ratio (PR) and 95% confidence intervals (CIs) were calculated. The predicted variables were selected following a stepwise regression method, and the best-adjusted model was based on the Akaike Information Criterion. The level of statistical significance was considered at a *p*-value of <0.05 for the primary endpoint and <0.025 for the secondary endpoint, with Bonferroni adjustments for two variables.

All analyses were performed using IBM SPSS v.26 (IBM Corp., Armonk, NY, USA) and the free software environment for statistical computing and graphics R v.4.0.2 [31].

## 3. Results

### 3.1. Characteristics of the Study Participants

In the study period, a total of 923 pregnant women were eligible for inclusion. Thirty-seven were excluded, for an entire sample of 886, although serum 25(OH)D levels were only obtained for 881. In Table 1, the participants’ characteristics are described in relation to GDM.

Among the study participants, the number of women with GDM accounted for 10.5% (95%CI = 8.6–12.7). The mean serum 25(OH)D concentration of the entire sample was 19.3 (8.9) ng/mL (95%CI = 18.7–19.9). In total, 55.5% (95%CI = 51.9–58.4) of the pregnant women presented with VDD. The mean age was 32.0 (5.8) years (95%CI = 31.6–32.4), and the mean BMI value was 24.8 (4.8) (95% CI = 24.5–25.1). The distribution of BMI into groups resulted in 60.4% normal weight (95%CI = 56.8–63.2), followed by 26.6% overweight (95%CI = 23.6–29.4) and 13.0% (95%CI = 10.9–15.4) obese. A maternal smoking habit was present in 10.3% (95% CI = 8.4–12.4) of the entire sample. Caucasian ethnicity represented 86.7% (95%CI = 84.0–88.6). Primigravida accounted for 50.3% (95%CI = 47.1–53.6), two pregnancies for 37.5% (95%CI = 34.3–40.7), and three or more pregnancies for 12.2% (95%CI = 10.2–14.5) of the sample. A previous cesarean section was found in 11.1% (95%CI = 9.2–13.3) of the participants.

**Figure 1:**
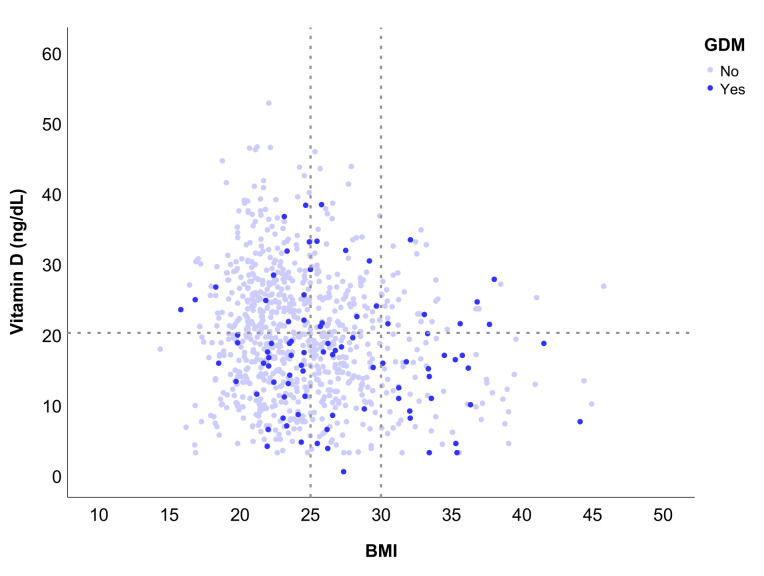
Individual vitamin D serum level distribution across the entire sample, in relation to BMI and GDM (the horizontal dotted line represents the threshold for VDD as <20 ng/dL, while the vertical dotted lines represent the limits of the BMI groups: Normal weight <25, overweight 25–30, and obesity >30).

Figure 1 depicts the distribution of the individual values of 25(OH)D, in relation to BMI, highlighting women with GDM. Horizontal dotted lines represent the limit for considering VDD, and vertical lines represent limits for the BMI groups. There was a high density of women with VDD and GDM, with a lower density of overweight or obese women with GDM.

In the univariate analysis (Table 1), the participants were compared according to the presence of GDM. The GDM group showed lower mean values for 25(OH)D (*p* = 0.021) and a higher prevalence for VDD (68.2% vs. 54.1%, *p* = 0.012). In addition, in the comparison of continuous variables, those pregnancies with GDM presented significantly older ages (*p* < 0.001), higher BMIs (*p* < 0.001), higher levels of SAP (*p* < 0.001) and DAP (*p* < 0.001) in the first trimester, lower levels of HDL cholesterol (*p* = 0.034), and higher levels of triglycerides (*p* < 0.001), C-reactive protein (*p* = 0.005), and fibrinogen (*p* < 0.001). In the qualitative analysis, the frequency of ethnicity was different, with Caucasians being more frequent in the non-GDM group (*p* = 0.045). In accordance with BMI, significant differences were observed, and the proportion of obese women was much higher in the GDM group (32.2% vs. 10.8%, *p* < 0.001). No significant differences were detected in the remaining variables.

**Figure 2:**
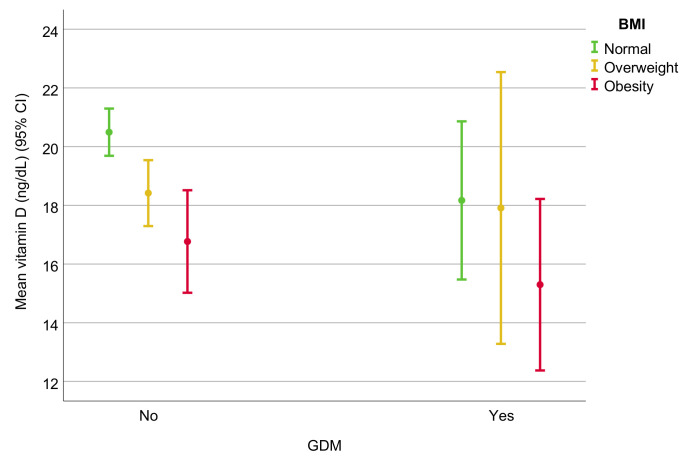
Mean vitamin D serum levels in relation to BMI and grouped by the presence of GDM. Error bars represent the mean and 95% confidence intervals.

Figure 2 represents the mean serum 25(OH)D levels in relation to the BMI group, grouped by the presence of GDM. In the absence of GDM, the 25(OH)D levels were significantly lower in the obesity group (CI95%, 15.2–18.5) than in the normal-weight group (CI95%, 19.6–21.3, *p* < 0.001). The trend was similar in the presence of GDM, although not achieving statistical significance (*p* = 0.376).

### 3.2. Assessment of the Association between VDD and GDM: Relationship with BMI

An association study between VDD and GDM was performed by multivariate analysis, using Poisson regression-adjusted models with robust variance. The prevalence ratio (PR) and 95% confidence intervals (CIs) were calculated (Table 2).

In the crude model, VDD was significantly associated with an increased prevalence of GDM (95%CI = 1.119–2.637; *p* = 0.013). Obesity was also associated with GDM (95%CI = 2.212–5.181; *p* < 0.001), but not solely overweight (95%CI = 0.744–2.001; *p* = 0.431). The other variables associated with GDM were older age (95%CI = 1.035–1.122; *p* < 0.001) and higher levels of triglycerides (95%CI = 1.004–1.007; *p* < 0.001), fibrinogen (95%CI = 1.005–1.011; *p* < 0.001), SAP (95%CI = 1.025–1.060; *p* < 0.001), and DAP (95%CI = 1.032–1.079; *p* < 0.001) in the first trimester and lower levels of HDL cholesterol (95%CI = 0.970–0.997; *p* = 0.019). Other ethnicities compared to Caucasian were also associated with GDM (95%CI = 1.169–3.665; *p* = 0.013).

There was no association with any of the other variables analyzed, noting the absence of an association with maternal variables such as smoking habit, history of hypothyroidism, or score in the physical activity, nutritional habits, or lifestyle items in the questionnaire.

In Model 1, which included exclusively VDD and BMI as an explanatory model of independent variables, both variables were significant. The prevalence ratio for VDD was 1.567 (95%CI = 1.016–2.417, *p* = 0.042), while for obesity, it was 2.992 (95%CI = 1.907–4.694; *p* < 0.001).

In Model 2, adjusted for all significant variables, the obese group lost its statistical significance. When fitted by age (*p* = 0.003), triglycerides (*p* = 0.002), fibrinogen (*p* = 0.001), and SAP in the first trimester (*p* = 0.003), the effect of obesity on GDM prevalence disappeared (PR = 1.395; 95%CI = 0.788–2.469, *p* = 0.253), while VDD maintained its significant association with GDM (PR = 1.660; 95%CI = 1.042–2.645; *p* = 0.033).

For construction of the adjusted model (AIC), a stepwise regression method was used, and variable selection was based on the Akaike Information Criterion. The presence of VDD was associated with a 1.6-fold increased risk of GDM (PR = 1.635; 95%CI = 1.027–2.604; *p* = 0.038) when adjusted by age (*p* = 0.003), triglycerides (*p* < 0.001), fibrinogen (*p* < 0.001), and SAP in the first trimester (*p* < 0.001).

This finding means that both VDD and obesity are associated with an increased prevalence for GDM, but once adjusted by other variables, only VDD retained its significant association with GDM.

### 3.3. Assessment of Secondary Obstetric and Neonatal Events: Association with VDD and BMI

Secondary obstetric and neonatal events were analyzed in relation to VDD and BMI (Table 3). In the case of VDD, there were no differences in event occurrence, apart from the increased percentage of GDM (12.3% vs. 7.1%; *p* = 0.012). With regard to BMI, GDM prevalence was much higher in the obese (26.1%) group, compared to the overweight (9.4%) and normal weight (7.7%) groups (*p* < 0.001). Overweight pregnant women presented a higher proportion of preterm births compared to normal weight and obese women, although this did not reach statistical significance. An increment in the frequency of births by cesarean section and newborns with a weight percentile >90 was observed as BMI increased. The proportion of cases with a head circumference percentile >90 was also higher but did not achieve statistical significance.

## 4. Discussion

Our results revealed that there was a high frequency of VDD in the pregnant women of our region (55.5%). In our daily work, between five and six pregnant women will present this condition. This finding is consistent with previously reported data [5,6].

The prevalence of GDM in our department is similar to the Spanish pregnant women’s population [16]. Roughly, 1 in 10 pregnancies receives this diagnosis, which becomes a relevant health problem.

In this study, we found that there were higher levels of VDD (68.2% vs. 54.1%) and obesity (32.2% vs 10.8%) in GDM pregnant women. In the multivariate analysis, we found a statistically significant PR of 1.635 for GDM when VDD was present. This association was not dependent on BMI. These outcomes are in line with recently reported results in the Taiwanese population, which revealed a nonlinear relationship between the 25(OH)D plasma levels and the risk of GDM [32]. The relationship between VDD and BMI and the potential effect of BMI has recently been explored in the Chinese population. A correlation has been described, being stronger in the overweight and obese groups [33].

As opposed to other studies [9,25], we did not detect VDD being associated with an increased frequency of secondary events, apart from GDM (12.3% vs. 7.1%). There were no differences in the frequencies of preterm birth, preeclampsia, and neonatal admission to ICU, or in the distribution of neonatal measurement percentiles. We ignored whether calcidiol supplementation in our VDD pregnant women could have influenced the incidence of these secondary events. Moreover, the observed frequencies of secondary events in our study were lower than those reported, except for GDM. Thus, there is a possibility that the differences were not detected.

However, we found differences in secondary events with regard to the BMI group. A nonsignificant higher frequency of preterm births was observed in the overweight group, but not in the obese group. These results differ from those of previous studies [34,35]. We propose that we did not find any association in obese women due to the small size of this subgroup and the low expected incidence. Both births by cesarean section and a neonatal weight percentile >90 were more prevalent in the obese group, but we did not detect any difference in neonatal ICU admissions as compared to the others [35].

The link between VDD and GDM enables us to focus on a population for which to take action. We suggest further longitudinal studies to establish any relation of causality. This could lead to intervention with vitamin D supplementation and a potential influence on GDM incidence, thereby potentially preventing future disorders such as diabetes mellitus and metabolic syndrome.

Some limitations should be recognized. First, due to the regional nature of this study, our results might not be generalizable. Additional studies are necessary to corroborate our findings in other locations around the world. Second, an important limitation of our investigation was its cross-sectional nature, which precluded a causality analysis. With this experimental design, the fact that GDM modifies 25(OH)D serum levels and all other data cannot be excluded. Another limitation could be the lockdown in Spain that started in March 2020 due to the COVID-19 pandemic. This special situation may have impacted the prevalence of VDD, since the major source of vitamin D for children and adults is exposure to natural sunlight. Thus, a major cause of VDD is inadequate exposure to sunlight [36]. The lockdown might have reduced sun exposure in the pregnant women population of our region. However, since this was not a variable in our study, it has not been analyzed. Finally, calcidiol supplementation in VDD participants, from the 26th week of pregnancy, could have biased the incidence of secondary maternal and neonatal events. Following national recommendation guidelines, it was considered unethical not to supplement with calcidiol, once a VDD pregnant woman had been identified. Nevertheless, it is uncertain if the administered doses achieved the normalization of the 25(OH)D serum concentration, as no further analyses were performed.

## 5. Conclusions

Our results revealed an association between VDD and GDM, which was independent of BMI. This relationship may indicate a possible influence of 25(OH)D in GDM development. However, further longitudinal studies are needed to establish causality.

VDD was not related to secondary events, in opposition to the BMI group, which showed a higher proportion of births by cesarean section and a neonatal weight percentile >90 in obesity.

## Figures and Tables

**Table 1:** Participants’ characteristics in relation to gestational diabetes mellitus (GDM).

Characteristics	Entire Sample	Non-GDM (*n* = 793, 89.5%)	GDM (*n* = 93, 10.5%)	*p*-Value
Sociodemographic characteristics				
Age (years), mean (SD)	32.0 (5.8)	31.7 (5.7)	34.2 (5.7)	<0.001 *
Questionnaire (St. Carlos Study), *n* (%)				
Physical activity	−0.7 (1.4)	−0.7 (1.4)	−0.8 (1.3)	0.942
Nutritional status	3.9 (3.7)	3.8 (3.7)	4.0 (3.6)	0.813
Lifestyle	3.1 (4.2)	3.2 (3.9)	3.2 (3.9)	0.758
Maternal smoking habit, *n* (%)	91 (10.3)	79 (10.0)	12 (12.9)	0.377
Maternal hypothyroidism, *n* (%)	48 (5.4)	40 (5.0)	8 (8.6)	0.152
Ethnicity, *n* (%)				0.045 *
Caucasian	766 (86.7)	692 (87.6)	74 (80.6)	
South American	62 (7.0)	54 (6.8)	8 (8.6)	
Other	55 (6.2)	44 (5.6)	11 (11.8)	
Pregnancy-related characteristics				
SAP, first trimester (mmHg), mean (SD)	110.8 (11.7)	110.2 (11.5)	116.4 (12.0)	<0.001 *
DAP, first trimester (mmHg), mean (SD)	68.8 (8.8)	68.3 (8.6)	73.0 (9.7)	<0.001 *
VDD, *n* (%)	489 (55.5)	429 (54.1)	60 (68.2)	0.012 *
BMI, *n* (%)	24.8 (4.8)	24.5 (4.5)	27.5 (5.9)	0.021 *
BMI group, *n* (%)				<0.001 *
Normal (<25)	532 (60.4)	491 (62.3)	41 (44.1)	
Overweight (25–30)	234 (26.6)	212 (26.9)	22 (23.7)	
Obesity (>30)	115 (13.0)	85 (10.8)	30 (32.2)	
Parity, *n* (%)				0.464
Primigravida	446 (50.3)	402 (50.7)	44 (47.3)	
2 pregnancies	332 (37.5)	298 (37.6)	34 (36.6)	
≥3 pregnancies	108 (12.2)	93 (11.7)	15 (16.1)	
History of cesarean section, *n* (%)	98 (11.1)	85 (10.5)	13 (14.0)	0.345
Gestational hypothyroidism, *n* (%)	195 (22.0)	175 (22.1)	20 (21.5)	0.901
Blood test, mean (SD)				
Vitamin D (ng/dL)	19.3 (8.9)	19.5 (8.9)	17.2 (8.6)	0.021 *
C-reactive protein (mg/dL)	5.7 (5.8)	5.7 (6.0)	6.2 (4.0)	0.005 *
Ferritin (mg/dL)	24.6 (25.5)	24.3 (25.8)	26.7 (21.7)	0.249
Cholesterol (mg/dL)	224.3 (38.7)	223.7 (38.8)	229.5 (37.4)	0.084
HDL cholesterol (mg/dL)	75.8 (15.3)	76.3 (15.2)	72.1 (15.7)	0.034 *
LDL cholesterol (mg/dL)	114.1 (32.5)	113.5 (32.0)	119.7 (36.1)	0.197
Triglycerides (mg/dL)	177.2 (68.1)	172.2 (62.4)	220.5 (95.3)	<0.001 *
Fibrinogen (mg/dL)	397.8 (56.3)	394.5 (54.6)	425.5 (62.3)	<0.001 *
Hemoglobin (g/dL)	11.6 (0.9)	11.6 (0.9)	11.6 (0.8)	0.747
Hematocrit (%)	34.2 (2.5)	34.2 (2.5)	34.2 (2.4)	0.597

* *p* < 0.05. Abbreviations: GDM, gestational diabetes mellitus; VDD, vitamin D deficiency; SAP, systolic arterial pressure; DAP, diastolic arterial pressure; BMI, body mass index.

**Table 2:** Association study. Prevalence ratio for GDM, estimated by Poisson regression models.

		Crude Model			Model 1			Model 2			Adjusted Model (AIC)		
		PR	95%CI	*p*-Value	PR	95%CI	*p*-Value	PR	95%CI	*p*-Value	PR	95%CI	*p*-Value
VDD:	No	1			1			1			1		
	Yes	1.718	(1.119–2.637)	0.013 *	1.567	(1.016–2.417)	0.042 *	1.660	(1.042–2.645)	0.033 *	1.635	(1.027–2.604)	0.038 *
BMI:	Normal	1			1			1					
	Overweight	1.220	(0.744–2.001)	0.431	1.137	(0.680–1.899)	0.625	0.724	(0.404–1.299)	0.279	NS		
	Obesity	3.385	(2.212–5.181)	<0.001 *	2.992	(1.907–4.694)	<0.001 *	1.395	(0.788–2.469)	0.253	NS		
Ethnicity:	Caucasian	1			-								
	South American	1.336	(0.675–2.642)	0.406	-			NS			NS		
	Other	2.070	(1.169–3.665)	0.013 *	-			NS			NS		
Parity:	Primigravida	1			-								
	2 pregnancies	1.038	(0.679–1.587)	0.863	-			NS			NS		
	≥3 pregnancies	1.408	(0.815–2.433)	0.220	-			NS			NS		
Previous cesarean section		1.305	(0.755–2.256)	0.340	-			NS			NS		
Gestational hypothyroidism		0.971	(0.608–1.551)	0.901	-			NS			NS		
Age		1.078	(1.035–1.122)	<0.001 *	-			1.062	(1.020–1.104)	0.003 *	1.064	(1.022–1.107)	0.003 *
Triglycerides		1.005	(1.004–1.007)	<0.001 *	-			1.003	(1.001–1.005)	0.002 *	1.003	(1.002–1.005)	<0.001 *
Fibrinogen		1.008	(1.005–1.011)	<0.001 *	-			1.006	(1.002–1.009)	0.001 *	1.005	(1.002–1.009)	<0.001 *
SAP		1.042	(1.025–1.060)	<0.001 *	-			1.027	(1.009–1.045)	0.003 *	1.031	(1.013–1.049)	<0.001 *
DAP		1.055	(1.032–1.079)	<0.001 *	-			NS			NS		
CRP		1.013	(0.994–1.032)	0.193	-			NS			NS		
Ferritin		1.003	(0.998–1.007)	0.285	-			NS			NS		
Cholesterol		1.003	(0.999–1.008)	0.160	-			NS			NS		
HDL		0.984	(0.970–0.997)	0.019 *	-			NS			NS		
LDL		1.005	(0.998–1.012)	0.133	-			NS			NS		
Questionnaire:	Physical activity	0.990	(0.858–1.142)	0.889	-			NS			NS		
	Nutritional habit	1.008	(0.951–1.069)	0.778	-			NS			NS		
	Lifestyle	1.005	(0.958–1.055)	0.827	-			NS			NS		
Hemoglobin		1.046	(0.837–1.308)	0.692	-			NS			NS		
Hematocrit		1.010	(0.939–1.087)	0.793	-			NS			NS		
Maternal smoking habit		1.294	(0.735–2.280)	0.372	-			NS			NS		
Maternal hypothyroidism		1.643	(0.846–3.192)	0.143	-			NS			NS		

* *p* < 0.05. Abbreviations: PR, prevalence ratio; CI, confidence interval; NS, non-significant; BMI, body mass index; VDD, vitamin D deficiency; SAP, systolic arterial pressure; DAP, diastolic arterial pressure; CRP, C-reactive protein. Model 1: Fitted exclusively with VDD and BMI. Model 2: Further adjusted with age, triglycerides, fibrinogen, and SAP. Adjusted model: Constructed following a stepwise regression methodology based on the Akaike Information Criterion.

**Table 3:** Secondary maternal and neonatal events: Associations with VDD and BMI.

	VDD		*p*-Value	BMI Group			*p*-Value
	No	Yes		Normal	Overweight	Obese	
GDM	28 (7.1)	60 (12.3)	0.012 *	41 (7.7)	22 (9.4)	30 (26.1)	<0.001 *
Preterm birth	23 (5.9)	20 (4.1)	0.224	23 (4.3)	18 (7.7)	2 (1.7)	0.034
Preeclampsia	10 (2.6)	13 (2.7)	0.921	11 (2.1)	6 (2.6)	6 (5.2)	0.158
Cesarean section	79 (20.3)	90 (18.6)	0.534	86 (16.3)	52 (22.5)	31 (27.4)	0.009 *
Neonatal ICU admission	79 (21.3)	99 (21.2)	0.961	106 (21.0)	42 (18.8)	32 (28.6)	0.115
Weight percentile			0.054				<0.001 *
<10	35 (8.9)	29 (5.9)		44 (8.3)	9 (3.8)	10 (8.7)	
10–90	312 (79.6)	377 (77.1)		424 (79.7)	188 (80.3)	75 (65.2)	
>90	30 (7.7)	59 (12.1)		39 (7.3)	27 (11.5)	25 (21.7)	
Missing	15 (3.8)	24 (4.9)		25 (4.7)	10 (4.3)	5 (4.3)	
Height percentile			0.068				0.078
<10	32 (8.2)	21 (4.3)		34 (6.4)	10 (4.3)	9 (7.8)	
10–90	272 (69.4)	349 (71.4)		386 (72.6)	164 (70.1)	69 (60.0)	
>90	71 (18.1)	88 (18.0)		85 (16.0)	44 (18.8)	31 (27.0)	
Missing	17 (4.3)	31 (6.3)		27 (5.1)	16 (6.8)	6 (5.2)	
Head circumference percentile			0.465				0.039
<10	31 (7.9)	43 (8.8)		50 (9.4)	12 (5.1)	12 (10.4)	
10–90	322 (82.1)	388 (79.3)		431 (81.0)	192 (82.1)	84 (73.0)	
>90	21 (5.4)	24 (4.9)		23 (4.3)	12 (5.1)	12 (10.4)	
Missing	18 (4.6)	34 (7.0)		28 (5.3)	18 (7.7)	7 (6.1)	

* *p* < 0.025. Abbreviations: GDM, gestational diabetes mellitus; VDD, vitamin D deficiency; ICU, intensive care unit.

## Data Availability

Not applicable.
